# Two new ArrayTrack libraries for personalized biomedical research

**DOI:** 10.1186/1471-2105-11-S6-S6

**Published:** 2010-10-07

**Authors:** Joshua Xu, Carolyn Wise, Vijayalakshmi Varma, Hong Fang, Baitang Ning, Huixiao Hong, Weida Tong, Jim Kaput

**Affiliations:** 1 Z-Tech Corporation, an ICF International company at NCTR, National Center for Toxicological Research, 3900 NCTR Rd., Jefferson, AR 72079, USA; 2 Division of Personalized Nutrition and Medicine, National Center for Toxicological Research, 3900 NCTR Rd., Jefferson, AR 72079, USA; 3 Center for Toxicoinformatics, Division of Systems Toxicology, National Center for Toxicological Research, 3900 NCTR Rd., Jefferson, AR 72079, USA

## Abstract

**Background:**

Recent advances in high-throughput genotyping technology are paving the way for research in personalized medicine and nutrition. However, most of the genetic markers identified from association studies account for a small contribution to the total risk/benefit of the studied phenotypic trait.  Testing whether the candidate genes identified by association studies are causal is critically important to the development of personalized medicine and nutrition. An efficient data mining strategy and a set of sophisticated tools are necessary to help better understand and utilize the findings from genetic association studies.

**Description:**

SNP (single nucleotide polymorphism) and QTL (quantitative trait locus) libraries were constructed and incorporated into ArrayTrack, with user-friendly interfaces and powerful search features. Data from several public repositories were collected in the SNP and QTL libraries and connected to other domain libraries (genes, proteins, metabolites, and pathways) in ArrayTrack.  Linking the data sets within ArrayTrack allows searching of SNP and QTL data as well as their relationships to other biological molecules. The SNP library includes approximately 15 million human SNPs and their annotations, while the QTL library contains publically available QTLs identified in mouse, rat, and human. The QTL library was developed for finding the overlap between the map position of a candidate or metabolic gene and QTLs from these species. Two use cases were included to demonstrate the utility of these tools. The SNP and QTL libraries are freely available to the public through ArrayTrack at http://www.fda.gov/ArrayTrack.

**Conclusions:**

These libraries developed in ArrayTrack contain comprehensive information on SNPs and QTLs and are further cross-linked to other libraries. Connecting domain specific knowledge is a cornerstone of systems biology strategies and allows for a better understanding of the genetic and biological context of the findings from genetic association studies.

## Background

Genetic variations are a major factor for inter-individual differences in disease susceptibility and response to environmental exposures such as nutrients and drugs. Recent advances in microarray-based genotyping techniques have enabled researchers to rapidly scan for known single nucleotide polymorphisms (SNPs), one of the most common genetic variations, across complete genomes.  Genome wide association studies (GWAS) have identified putative variations that contribute to common, complex diseases such as asthma, cancer, diabetes, heart disease and mental illnesses. SNPs that have been associated with complex diseases may eventually be used to develop better strategies to detect, treat and prevent these diseases. A web-based catalog of GWAS publications has been created and periodically updated at the National Human Genome Research Institute [1].  Such technology is contributing to the development of personalized medicine, in which the current one-size-fits-all approach to medical care will give way to more customized treatment strategies.  

However, it is uncommon for GWAS to incorporate diet or environmental exposures which are known to influence disease susceptibility ([2,3] and http://www.nugo.org/nutrialerts/39848).  In addition, many GWAS have been done in European populations and their applicability to other populations and individuals has not been adequately studied ([4-6] and http://www.nugo.org/nutrialerts/40314 and http://www.nugo.org/nutrialerts/38373).  GWAS results must therefore be further tested to determine whether the statistical associations found offer real-world potential to predict complex phenotypes or are useful in developing testable hypotheses about the development, progression, or treatment of a disease.  

A novel strategy has been proposed to analyze gene-nutrient interactions, aiming to discover genes that contribute to individual risk factors [7-9]. This data mining strategy is based on analyzing candidate genes involved in nutrient metabolism or regulation and mapping those genes to quantitative trait loci (QTL) contributing to a particular trait or condition.  A QTL is a region of DNA that is associated with a particular phenotypic trait. A common use of QTL data is to identify candidate genes underlying a trait within one or more QTL. This approach utilizes the available genomic, physiological, and environmental data to select candidate genes for further analyses.  

A limitation of this type of strategy is that many databases are knowledge or domain specific – that is, they limit data to one discipline such as proteomics, genomics, or metabolomics.  To address this limitation, we propose a solution through ArrayTrack. ArrayTrack is a publicly accessible microarray data management and analysis system developed by the FDA’s National Center for Toxicological Research [10,11]. It has been extended to manage and analyze preprocessed proteomics and metabolomics experiment data. To facilitate data interpretation, ArrayTrack has integrated a rich collection of biological information for genes, proteins and pathways, which are drawn from public repositories and organized as individual yet cross-linked libraries. Thus it provides a one-stop solution for omics data analysis and interpretation in the context of gene-function relationship. 

One of the focuses in GWAS is to relate SNPs to genes and pathways to understand the underlying mechanisms of the studied disease. The SNP-gene-pathway relationship should be dynamically interrogated in an interactive/integrated environment. ArrayTrack has provided a gene-pathway exploratory platform. By integrating the SNP library that contains annotation summary information of SNPs and their mapped relationship to genes, ArrayTrack now enables dynamic analysis of the SNP-gene-pathway relationship and thus offers support to SNP studies. The identification of the SNP-gene-QTL relationship is the basis to test whether the gene/SNP is associated with the etiology of a disease in animal models or human studies. The integration of SNP and QTL libraries into ArrayTrack enables dynamic mining of such complex biological interactions and thus expands the utility of ArrayTrack.

## Construction and content

A major goal of the SNP and QTL libraries is to collect dispersed data in one place, allowing researchers to easily access and compare data across multiple knowledge bases. Data have been downloaded from public repositories and reorganized as library components of ArrayTrack. The data in the SNP and QTL libraries can directly link back to their sources, as well as ArrayTrack’s own existing collection of libraries.

### SNP library

Data for the SNP library with annotation summary information were downloaded to ArrayTrack (an Oracle Enterprise Edition 10g database) from the UCSC Genome Bioinformatics Site[12] and the NCBI dbSNP[13].  This guarantees a seamless external connection to its Genome Browser[12] for each SNP with a link constructed based on the SNP’s chromosomal position. The UCSC Genome Bioinformatics Site reports different positions than the NCBI dbSNP database for a small subset of SNPs. The annotation summary information is organized as one database table (Table 1). The SNP library includes approximately 15 million human SNPs and their annotations.

**Table 1:** Data field names and description of the SNP annotation summary database table.

*Field Name*	*Field Description*
chrom	Chromosome identifier
BP_Start	Physical location start position in chromosome
BP_End	Physical location end position in chromosome
name	Reference SNP identifier (rs#)
strand	DNA strand (+/-) containing the observed alleles
observed	The sequences of the observed alleles
molType	Sample type from exemplar submission
class	The class of variant (single, in-del, insertion, microsatellite, etc)
valid	The validation status of the SNP
avHet	The average heterozygosity from all observations
avHetSE	The Standard Error for the average heterozygosity
func	The functional category (intron, synonymous, missense, etc)
locType	How the variant affects the reference sequence

For additional annotations, external links are provided for each SNP to the websites of dbSNP, UCSC Genome Browser, Ensembl[14], and the International HapMap Project [15-17]. These websites provide information about SNP allele frequency distributions among different populations, linkage with nearby genetic variants, functional annotations, and pathways involving the related genes [18,19].  Major online SNP databases and resources are listed at http://www.nugo.org/nutrialerts/40615. The SNP library also maps SNPs to genes in ArrayTrack’s Gene library based on the relationships downloaded from dbSNP.

### QTL library

For the QTL library, data for mouse, rat, and human QTLs were collected from species-specific databases.  QTL data for mouse were taken from the Mouse Genome Database (MGI) at Jackson Laboratory[20]. Only those QTLs with a valid mapping position and an official validation status were imported. QTL data for rat and human were extracted from Rat Genome Database (RGD) developed by the Medical College of Wisconsin[21]. The processing of QTL data taken from RGD was much more complex due to a disagreement with the QTL position assignment method adopted by RGD.  A QTL in RGD is positioned on a genome assembly by using the flanking and peak markers as provided by the publication detailing the QTL. When only one flanking or peak marker is available, the QTL position is assigned using the QTL size estimates made from the global distribution of QTL sizes, which are 26 Mbp (million base pairs) for human and 45 Mbp for rat.  Many researchers would prefer to estimate these differences rather than rely on the default parameters. We excluded those QTLs that are identified by only one flanking marker since the confidence in QTL positions is quite low.  For those QTLs identified by the peak marker, the position of its peak marker was assigned to the associated QTL, without any estimate of the QTL’s size. Marker positions were pulled from RGD except for those markers that are actually genes, in which case gene positions from NCBI were used. Finally QTL data from all three species were stored together in one database table (Table 2).  Web links to the original data sources have been provided for detailed information about each QTL. Additionally, chromosome positions for all genes in human, rat, and mouse were downloaded from the NCBI ftp site and organized into a separate table to enable the cross-table query of genes and QTLs based on their map positions.  

**Table 2:** Data field names and description of the QTL annotation database table.

*Field Name*	*Field Description*
Tax_ID	Species taxonomy ID
External_ID	QTL’s ID assigned by the data source
Entrez_Gene_ID	QTL’s gene ID assigned by NCBI
Symbol	QTL representation symbol (short name)
QTL_Name	QTL full name (long description)
Chromosome	The chromosome that the QTL is positioned on
Strand	The chromosome strand that the QTL is positioned on
cM_Position	Estimated centiMorgan (cM) position on the chromosome
Chr_Start	Base pair starting position on the chromosome
Chr_End	Base pair ending position on the chromosome
Pos_Method	How position is determined
Ref_Pubmed	PubMed IDs for the original papers detailing the QTL
Synonyms	Other symbols may have been used
Phenotypes	Phenotype ontology annotation
Candidate Genes	Candidate genes mentioned by original papers

## Utility and discussion

**Figure 1:**
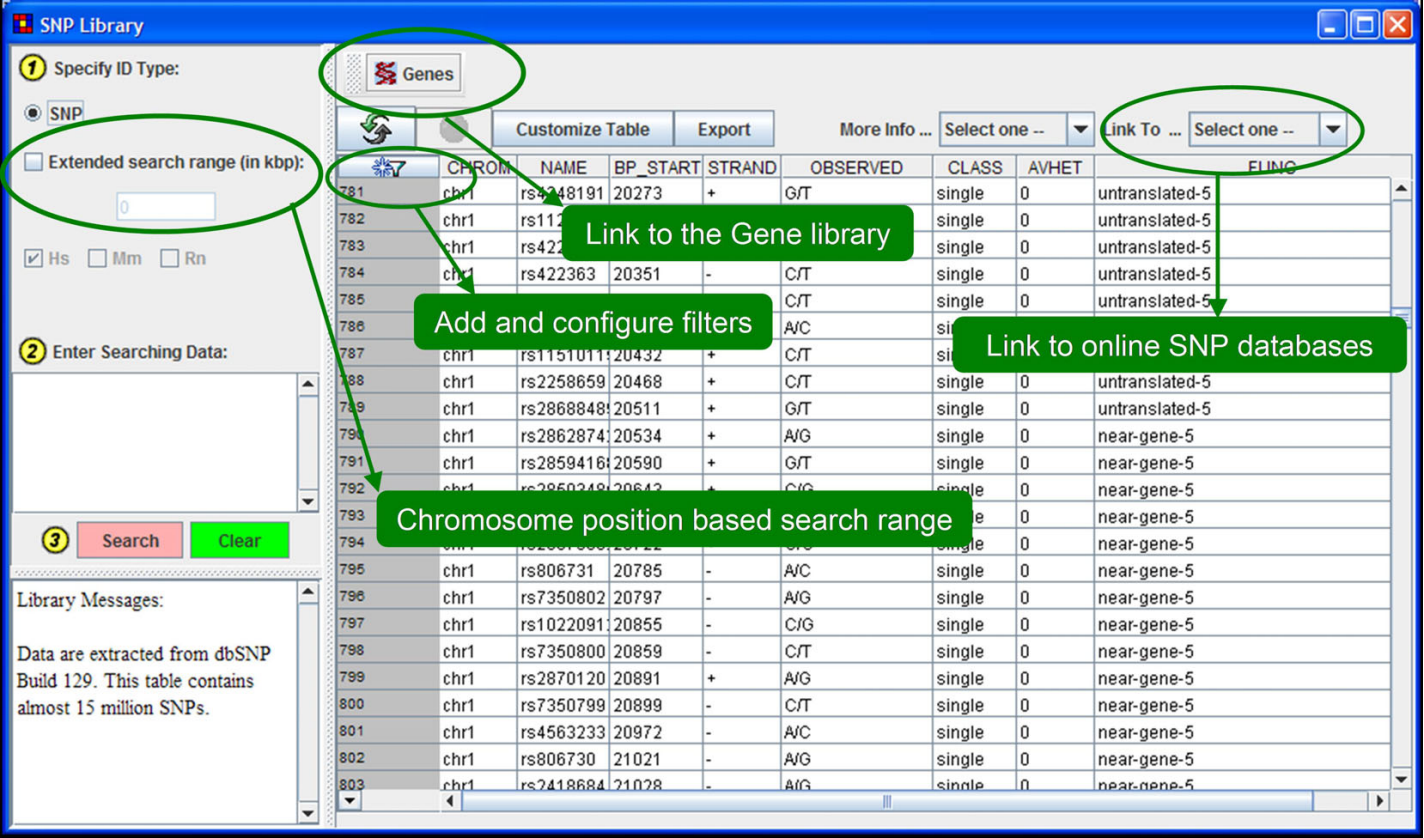
**The graphic view and query interface for the SNP library.** The left panel takes a list of SNPs as the query input and lets the user specify the extended search range. The species selection is not enabled as the SNP library contains only human SNPs. The top panel provides various functions such as mapping selected SNPs to genes, customizing the selection of data columns for display, exporting SNPs as spreadsheet or plain text, linking the selected SNP to external online databases, and complex filters configuration. The center panel displays the query results.

**Figure 2:**
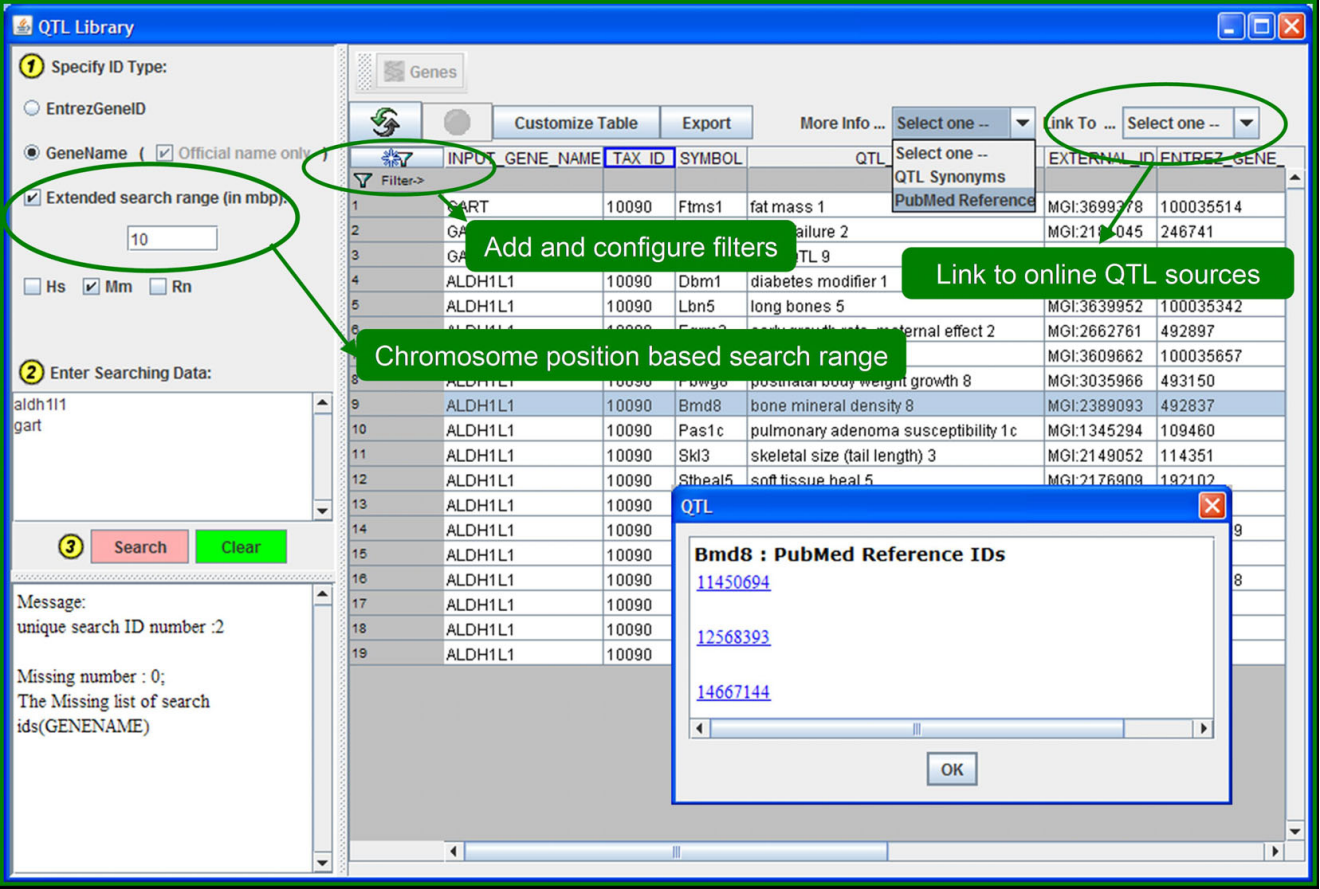
**The graphic view and query interface for the QTL library.** The left panel takes a list of genes as the query input and lets the user specify the extended search range. Genes may be specified with either Entrez gene ID or gene name. One or more species may be selected for querying QTLs. Hs, Mm, and Rn stand for human, mouse, and rat, respectively. The top panel provides various functions such as customizing the selection of data columns for display, exporting QTLs as a spreadsheet or plain text, linking the selected QTL to external online databases, listing QTL synonyms and related PubMed references, and complex filters configuration. The center panel displays the query results. The overlaid small panel shows PubMed Reference IDs for the selected QTL.

Many databases are cumbersome and difficult to browse or search. For example, only one SNP at a time may be queried and viewed in the well-designed and cross-linked SNP database at the dbSNP. Besides collecting dispersed data in one place to facilitate data mining across multiple knowledge domains, ArrayTrack also aims to facilitate accessibility of data. The SNP library (Figure 1) and QTL library (Figure 2) use a clean interface that offers a spreadsheet-like view of search results. Searches are very quick and offer comprehensive functionality that includes: extended mapping ranges, exact or partial matches, and combinations of query filters on all data fields. The addition of the SNP and QTL libraries to ArrayTrack opens up several new research opportunities. Following are two case studies of data mining strategies.

### *Gene – nutrient interaction*

This strategy was proposed to analyze gene-nutrient interactions, aiming to discover genes that contribute to risk factors that include environmental exposures[9]. Manual processes to find the QTLs that have map positions nearby those of each gene in a given list are labor intensive and time consuming. The QTL library completely automates this data mining process by searching and collecting data and providing a convenient list-based search interface. The strategy is comprised of two steps: 

1. Search the metabolic and regulatory pathways of a chosen nutrient to generate a list of genes regulated by or involved in the metabolism of such a nutrient.  Examples used to develop this approach included thiamine, folic acid, riboflavin, glucose, fructose, vitamin A, vitamin D, and vitamin E. The pathway for each gene or metabolite is searched individually. This step may be accomplished through GeneGo or other similar pathway search tools. 

2. Using the QTL library, map each gene to QTLs contributing to a phenotype.  In this case, the metabolic genes were “mapped” to QTLs for obesity, T2DM, body weight, or other related phenotypes or to QTLs that contribute to those diseases (for example, insulin or glucose level QTLs). The chromosomal position of each gene is found with the specified species mapping information and then used to construct a chromosomal search region for QTLs with a user specified range of extension.

An example of this strategy is shown in Table 3.  The dietary carbohydrate, fructose, is implicated in the pathogenesis of obesity, insulin resistance and cardiovascular diseases [22]. In order to identify the genes of the fructose metabolic pathway that are potentially underlie these disease processes, a pathway analyses program, GeneGo, was used. A total of 34 genes were acquired for fructose metabolism (rodent version), from the GeneGo “organism specific pathway map,” under carbohydrate metabolism. The genes obtained were uploaded on to ArrayTrack’s QTL library and searched for associations to QTLs choosing specificity for the mouse species.  The search range was set at 5 Mbp (million base pair). The 34 genes involved in fructose metabolism associated with a total of 108 QTLs. These results were filtered to retain only the QTLs that related to obesity, type 2 diabetes and cardiovascular diseases. Using this approach, 11 genes of the fructose metabolic pathway were identified to be associated to 19 mouse QTLs as depicted in Table 3.

**Table 3:** Fructose metabolic pathway genes mapped to QTLs related to obesity, type 2 diabetes and cardiovascular diseases.

GENE NAME	QTL_NAME	QTL SYMBOL	CHROMOSOME	CHR_START	CHR_END
ALDOB	atherosclerosis 8	Ath8	4	45671314	104972403
GALM	free fatty acid level 1	Ffal1	17	82306849	82306984
HK2	body weight QTL 18	Bw18	6	83663863	83664025
HK2	epididymal fat weight	Efw	6	75494350	94175949
HK3	predicted fat percentage 3	Pfat3	13	53042863	53042973
KHK	HDL QTL 22	Hdlq22	5	32132687	32132904
PFKFB4	body weight, QTL 6	Bwq6	9	110665094	110665094
PFKFB4	dietary obesity 2	Dob2	9	108316210	108316344
PFKM	HDL QTL 4	Hdlq4	15	96095961	96096089
PMM1	diabetes susceptibility QTL 2	Dbsq2	15	84216927	84217076
PMM1	HDL QTL 27	Hdlq27	15	81032515	81032665
PMM1	induction of brown adipocytes 8	Iba8	15	84216927	84217076
SLC37A4	HDL QTL 17	Hdlq17	9	43693207	43693338
SLC37A4	type 2 diabetes modifying QTL 1	Tdmq1	9	46041438	46041650
SORD	blood glucose level 1	Bglu1	2	117938185	174308571
SORD	multigenic obesity 5	Mob5	2	65277459	162502742
SORD	organ weight QTL 3	Orgwq3	2	123215789	123215931
SORD	type 2 diabetes mellitus 2 in SMXA RI mice	T2dm2sa	2	29273455	148358750
TPI1	atherosclerosis 37	Ath37	6	116614915	128469043

### *Connecting GWAS results with QTLs*

Both GWAS and QTL analyses associate a certain trait with genetic map positions. GWAS typically use unrelated populations of cases and controls.  QTL mapping studies are usually performed on inbred strains of animals or nuclear families (e.g., trios design) of humans. Combining GWAS results from human association studies with QTLs which are usually from laboratory animals increases the reliability of identifying candidate genes for further fine mapping studies, e.g., through next generation sequencing.   Primates and rodents have shared synteny, the co-localization of genes within a chromosomal region.  These shared chromosomal regions can be re-ordered within and among chromosomes between species, but their map positions have been well characterized (see NCBI MapView - http://preview.ncbi.nlm.nih.gov/mapview/). This strategy is comprised of three steps: 

1. Obtain a list of trait-associated SNPs from published GWAS results for a chosen condition such as obesity, T2DM, or hypertension. This can be quickly accomplished through querying the GWAS Catalog[1]. 

2. Using ArrayTrack’s SNP library, map each SNP to genes based on chromosomal positions. The result of this step is a list of genes.

3. For each gene in the list, query ArrayTrack’s QTL library to find whether there are any nearby QTLs that may contribute to the studied condition.  

As an example, a list of SNPs was obtained from the GWAS Catalog[1] that are associated with hypertension-related phenotypes such as elevated systolic or diastolic or both blood pressures, hypertension, and stroke. These SNPs were then mapped to human genes through the SNP library. Finally we searched the QTL library for those in human that, by mapping position, are close to any gene in the list. An extended search range of 2 Mbp (million base pair) was chosen and the results were filtered to keep those QTLs relevant to hypertension related traits or phenotypes. The final results are shown is Table 4. The genes identified by this strategy are candidates for further fine mapping in linkage or association studies and may be used to design animal studies to test their role in the mechanisms of hypertension. 

**Table 4:** Comparison of hypertension related GWAS findings and QTLs in humans. SYMBOL and CHR stands for QTL symbol and chromosome, respectively.

GWAS Gene	SYMBOL	QTL_NAME	EXTERNAL_ID	CHR	CHR_START	CHR_END	PUBMED_REF
CNTN4	BP30_H	Blood pressure QTL 30 (human)	RGD:1298439	3	1	32140379	10330357
CSK	BP32_H	Blood pressure QTL 32 (human)	RGD:1298444	15	24973428	1E+08	10330357
CDH13	BP33_H	Blood pressure QTL 33 (human)	RGD:1298498	16	12046946	87452910	10330357
PLCD3	BP34_H	Blood pressure QTL 34 (human)	RGD:1298468	17	569917	64059498	10330357
ZNF652	BP34_H	Blood pressure QTL 34 (human)	RGD:1298468	17	569917	64059498	10330357
ZNF652	BP35_H	Blood pressure QTL 35 (human)	RGD:1298421	17	34106437	65977271	9328471
PLCD3	BP35_H	Blood pressure QTL 35 (human)	RGD:1298421	17	34106437	65977271	9328471
STK39	BP46_H	Blood pressure QTL 46 (human)	RGD:1300023	2	75683889	218285001	11034945
CACNB2	BP6_H	Blood pressure QTL 6 (human)	RGD:1298448	10	6761916	19478134	12559686
PLCD3	BP89_H	Blood pressure QTL 89 (human)	RGD:1643075	17	36247937	70192683	16172425
ZNF652	BP89_H	Blood pressure QTL 89 (human)	RGD:1643075	17	36247937	70192683	16172425
MTHFR	BP9_H	Blood pressure QTL 9 (human)	RGD:1298463	1	12149647	12191864	10942422
CACNB2	FBRL4_H	Fibrinogen level QTL 4 (human)	RGD:1559246	10	18761298	99475525	12877910
C10ORF107	FBRL4_H	Fibrinogen level QTL 4 (human)	RGD:1559246	10	18761298	99475525	12877910
CACNB2	FBRL5_H	Fibrinogen level QTL 5 (human)	RGD:1559249	10	18761298	99475525	12877910
C10ORF107	FBRL5_H	Fibrinogen level QTL 5 (human)	RGD:1559249	10	18761298	99475525	12877910
ZNF652	FBRL6_H	Fibrinogen level QTL 6 (human)	RGD:1559242	17	10800103	77846317	12877910
PLCD3	FBRL6_H	Fibrinogen level QTL 6 (human)	RGD:1559242	17	10800103	77846317	12877910
CAMK4	HRTRT7_H	Heart rate QTL 7 (human)	RGD:1558696	5	110064214	110064525	12189495
STK39	HRV2_H	Heart rate variability QTL 2 (human)	RGD:1558699	2	59145280	208433251	12480036

Besides meeting the need of SNP interpretation and exploration, the integration of the SNP library with ArrayTrack’s library collection enables users to quickly explore and compare the associated biological pathways for SNPs of interest. Along with ArrayTrack’s library collection, the SNP and QTL libraries will be maintained and periodically updated as new data become available. As the development of these libraries progresses, query based on gene names will be added to the SNP library and query based on QTL symbols will be implemented for the QTL library.

## Conclusions

The massive amount of data generated in biomedical research studies is often considered and organized as separate knowledge domains.  We are developing strategies and tools such as the SNP and QTL libraries for data mining that will allow for more targeted research studies for developing the path to personalized nutrition, medicine, and healthcare.

## Availability and requirements

The SNP and QTL libraries are freely available to the public through ArrayTrack at http://www.fda.gov/ArrayTrack. 

## Competing interests

The authors declare that they have no competing interests.

## Authors’ contributions

CW and JK conceived the integration of the QTL library and related data mining strategies. HF and WT conceived the integration of the SNP library and its applications. VV, CW, JX, and JK developed the case studies. BN, HH, and HF suggested functions to be implemented with the libraries and helped with testing. JX developed the databases and software. JX created the first draft manuscript. All authors helped draft the manuscript and approved the final version.
